# Distinction of nucleobases – a tip-enhanced Raman approach

**DOI:** 10.3762/bjnano.2.66

**Published:** 2011-09-23

**Authors:** Regina Treffer, Xiumei Lin, Elena Bailo, Tanja Deckert-Gaudig, Volker Deckert

**Affiliations:** 1 IPHT – Institute for Photonic Technology, Albert-Einstein-Str. 9, D-07745 Jena, Germany; 2 WITec Wissenschaftliche Instrumente und Technologie GmbH, Lise-Meitner-Str. 6, D-89081 Ulm, Germany; 3 Institute of Physical Chemistry, Jena University, Helmholtzweg 4, D-07743 Jena, Germany

**Keywords:** DNA, nanoscale analysis, Raman, sequencing, TERS

## Abstract

The development of novel DNA sequencing methods is one of the ongoing challenges in various fields of research seeking to address the demand for sequence information. However, many of these techniques rely on some kind of labeling or amplification steps. Here we investigate the intrinsic properties of tip-enhanced Raman scattering (TERS) towards the development of a novel, label-free, direct sequencing method. It is known that TERS allows the acquisition of spectral information with high lateral resolution and single-molecule sensitivity. In the presented experiments, single stranded adenine and uracil homopolymers were immobilized on different kinds of substrates (mica and gold nanoplates) and TERS experiments were conducted, which demonstrated the reproducibility of the technique. To elucidate the signal contributions from the specific nucleobases, TERS spectra were collected on single stranded calf thymus DNA with arbitrary sequence. The results show that, while the Raman signals with respect to the four nucleobases differ remarkably, specific markers can be determined for each respective base. The combination of sensitivity and reproducibility shows that the crucial demands for a sequencing procedure are met.

## Introduction

The determination of the exact nucleobase sequence of DNA is of great importance for research in the life sciences. The first sequencing methods were published in 1977 by Maxam and Gilbert [1], and Sanger et al. [2]. Since then the sequencing technology has been refined and automated, and current advances show a tendency towards single-molecule sequencing, which eventually results in the development of sequencing systems with reasonable costs and expenditure of time [3].

Tip-enhanced Raman scattering (TERS) is the combination of Raman spectroscopy with a scanning probe microscope, most often an atomic force microscope (AFM). A metal nanoparticle at the apex of the AFM tip leads to a large enhancement of the electromagnetic field in the vicinity of the particle and consequently an increase of the Raman signal. Due to the size of the particle a lateral resolution of <20 nm can be reached [4–8]. The feasibility of TERS for biological samples has been exemplified by studies of specific molecules [9], single virus investigation [10] and studies of bacterial [11] and cellular systems [12–13], as well as of erythrocytes [14]. The first TERS experiments on DNA base nanocrystals [15–17] and a study of the hydrogen bonding between adenine and thymine on a gold substrate [18] showed that a distinction of the respective nucleobases is possible, and this eventually led to successful TERS measurements on a single RNA strand of a cytosine homopolymer [19].

The dependency of the electromagnetic field enhancement of TERS on the composition of the substrate, amongst other parameters, was shown in three-dimensional finite-difference time domain (3D-FDTD) simulations [20]. A metal substrate such as gold provides an additional field enhancement as it produces a large electromagnetic (EM) coupling with the tip, which is often called a “gap mode”. In contrast, dielectric materials cannot couple as effectively, and hence in this case the effect relies on an isolated tip, resulting in a much smaller enhancement [20–21]. Prerequisites for a metal substrate suitable for TERS experiments on single stranded DNA or RNA are an almost atomic flatness of the surface and, as the used TERS setup is operated in back-reflection mode (i.e., through the substrate and back), transparency, i.e., the substrate has to be sufficiently thin. A suitable approach is the synthesis of triangular and hexagonal flat and transparent gold [22] or silver [23] nanoparticles that are large enough to avoid specific localized enhancement regions.

The experiments described here probed the factors affecting the TERS spectra collected on single stranded adenine and uracil homopolymers immobilized on a mica surface (adenine) and on a gold nanoplate (uracil), with respect to the intensity and reproducibility.

Another challenge with regards to a sequencing procedure with TERS is the fact that the four nucleobases reveal remarkably different Raman scattering cross sections. It was shown that in SERS experiments on an equimolar mixture of the bases, the intensities of the ring breathing modes of the distinct bases are as follows: Poly-adenine > poly-cytosine >> poly-guanine > poly-thymine [24]. Furthermore, in a comparison of the SERS spectra of two DNA molecules with different adenine contents (15.5% and 44.3%), the adenine signals dominated [25]. Hence it was predicted that signals from adenine only are likely to be detected when performing TERS experiments on native DNA [14]. In contrast, TERS measurements on calf thymus DNA presented here clearly show that specific contributions of all four nucleobases can be determined. Hence, with the chosen samples poly(A), poly(U) and calf thymus DNA typical molecules covering DNA, RNA and single stranded DNA molecules containing all four bases were addressed.

## Results and Discussion

**Figure 1:**
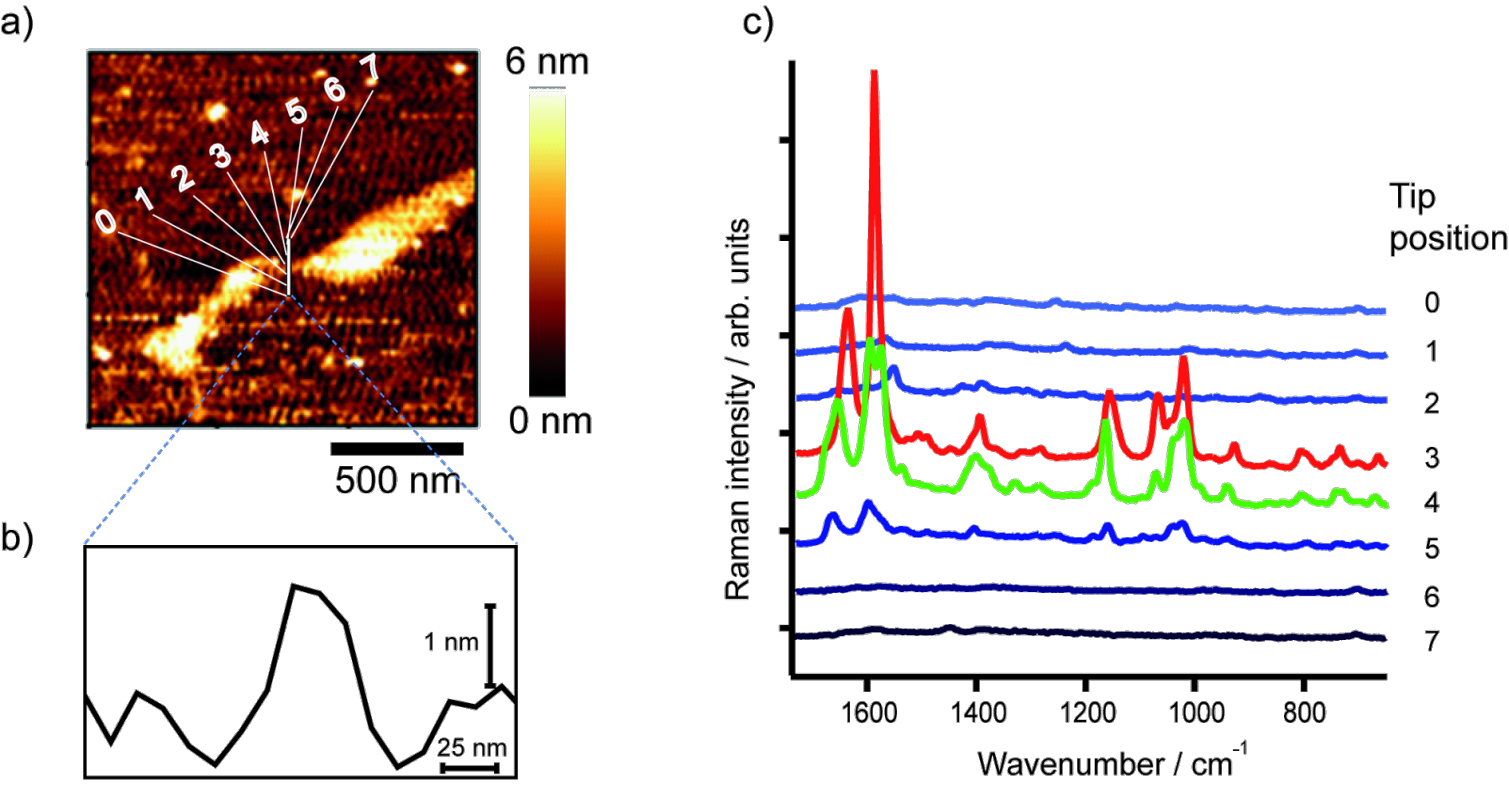
a) AFM topography image of adenine homopolymer, b) topography cross section of the raw data corresponding to the line indicated in a), and c) TERS spectra measured at the positions indicated in a). The AFM topography in a) was slightly smoothed (Gaussian) to enhance the contrast. The TERS experiment positions were selected using the topography as a template, but with a much smaller actual step size (10 nm) as it appears in the figure.

Figure 1a shows the AFM topography image of an adenine homopolymer on a mica substrate. As the height in the cross section is slightly higher than the height of a single DNA strand it is assumed that the sample is either coiled or that several strands are coagulated. TERS spectra were measured at eight different positions across the DNA each separated by a distance of 10 nm.

The measured positions are indicated in Figure 1a, and in Figure 1c the corresponding spectra are shown. Spectral features of poly(dA) can be seen at three positions, but only two exhibit a high enhancement, thus providing information on the enhancement area of the TERS tip. As the distance between two adjacent points is 10 nm, an enhancement region of <20 nm can be safely assumed. This value is a conservative estimation and in agreement with a tip diameter of approximately 20 nm, as we find typically from SEM pictures of similar probes [26].

Both spectra show bands that can be assigned to adenine and additional signals of the DNA backbone consisting of phosphate and ribose moieties. This would be not surprising in a normal Raman experiment, but in a TERS experiment the field decays rapidly with respect to the tip surface and these groups should only weakly contribute to the spectrum. Hence, the appearance of these signals could indicate that the DNA strands are not rigidly orientated on the mica substrate and, in addition to the previously mentioned topography arguments, support the assumption of a coiled or coagulated sample.

**Figure 2:**
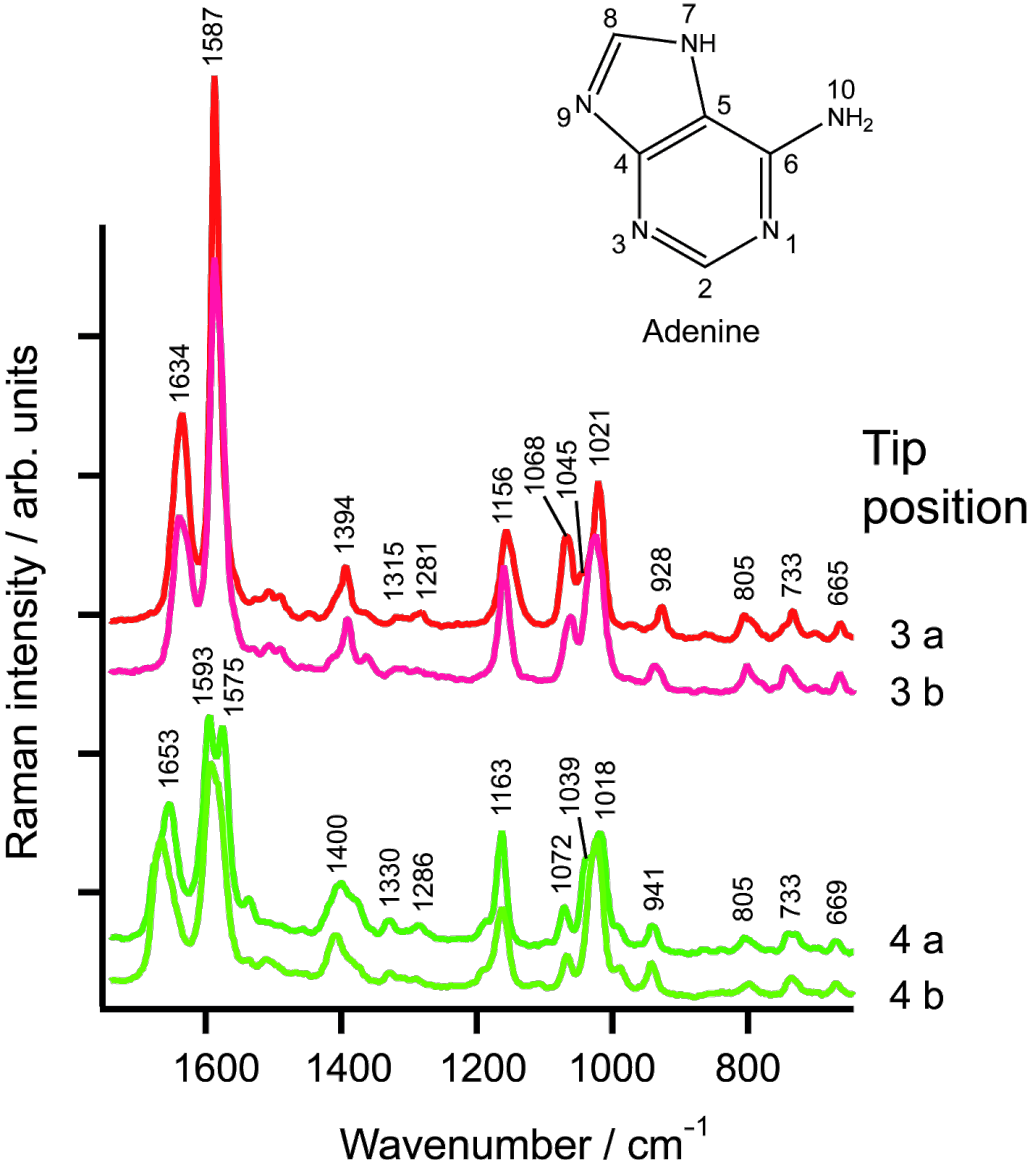
TERS spectra of the adenine homopolymer at positions 3 and 4 as indicated in Figure 1a. Both positions were measured twice (a, b).

Simultaneously the instrumental reproducibility was evaluated by measuring TERS spectra twice at the same position. The respective spectra are shown in Figure 2 indicating that these spectra are nearly identical. This clearly indicates that the tip was positioned reliably at the selected positions and that sample drift was negligible during the measurement. The results also suggest that minute changes of the tip position can affect the spectral appearance as different parts of the molecule/strand experience a different enhancement when the tip is slightly changing its position. These changes, as can be seen in Figure 2, mainly manifest as changes in the intensity or peak ratios, nevertheless an assignment is still possible. The even smaller variations at the same tip position, e.g., the splitting of the band 1590 cm^−1^ (Figure 2, tip position 4a and 4b), must then be attributed either to a drift of the instrument in the subnanometre range or to molecular orientation changes, e.g., rotation.

An assignment for the TERS spectra measured at positions 3 and 4 is given in Table 1. For the band assignment the spectral features were compared with those of SERS and Raman spectra of adenine derivates (deoxyadenosine (dA) [27], deoxyadenosine monophosphate (AMP) [28] and single stranded adenine homopolymer (poly(dA)) [29]).

**Table 1:** Band assignment of the TERS spectra of the adenine homopolymer (in cm^−1^).^a^

Mode	3	4	SERS [27]	NRS [27]	SERS [28]	NRS [29]	Assignment

1	665	669				663	ring def [29]
2	733	733	728	734	732	727	ring breathing (Py) [27,29]
3	805	805				790/819	bk (OPO str), ring [29]
4						842	bk (OPO str) [29]
5	928	941	919	908	(960)	917	NH_2_ rk [27], sugar [29]
6	1021	1018				1007	NH_2_ def
7	1045	1039	1035			1052	N–sugar str [27], sugar (CO str) [29]
8	1068	1072		1066		1092	N–sugar str [27], bk (PO_2_^−^ str) [29]
9	1156	1163	1171	1174		1163	(C_5_–C_6_) str [27]
10						1204	ring [29]
11						1221	ring [29]
12	1281	1286			1264	1251	ring [29]
13	1315	1330	1320	1348	1334	1306	C–N str [27], ring [29]
14						1336	ring [29]
15						1345	ring [29]
16	1394	1400	1389	1380	1370 1390	1378	(C_6_–N_1_) str (Py) [27], ring [29]
17						1423	ring [29]
18						1444	CH_2_ def [29]
19			1472	1478	1460	1462	C=N str (Py) [27], C_2_H_2_ def [29]
20						1485	ring [29]
21						1509	ring [29]
22	1587	1575	1551	1572		1581	ring str [27,29], NH_2_ def [29]
23		1593	1594				NH_2_ def [27]
24	1634	1653	1657				NH_2_ sci [27]

^a^Abbreviations: NRS, normal Raman scattering; SERS, surface-enhanced Raman scattering; Py, pyrimidine; bk, backbone; str, stretching; def, deformation; rk, rocking; sci, scissoring.

A comparison of TERS and SERS spectra of adenine (or any other molecule) must take into account that a SERS experiment is either done in a colloidal solution or the dissolved sample is brought into contact with a solid SERS substrate. In both cases the molecules can select favoured binding sites to adsorb to the metal particles. In case of adenine these are the four nitrogen atoms: The pyrimidine N_1_ and N_3_, the imidazole N_7_ and the N_10_ of the exocyclic NH_2_ group. N_9_ is the binding site to the ribose and therefore cannot bind to the metal nanoparticle. Density functional theory (DFT) calculations show changes in Raman band intensities and positions for the four respective complexes of adenine and a silver atom. These variations are attributed to a deformation of the adenine molecules to an energetically optimized conformation [16]. For the TERS experiments the adenine homopolymer was immobilized on a mica substrate and could not move freely. Consequently the TERS tip can be positioned at thermodynamically “unfavoured” sites, giving rise to a slightly different appearance of the spectra, in particular when chemical interactions between the tip and the SERS/TERS probe take place. Assuming an optimal immobilization, the phosphate backbone is fixed to the substrate and the bases should face upward. As single stranded DNA is a very flexible molecule it tends to form loops or twists along the strand, or even secondary helix structures. In this case the backbone becomes accessible and spectral contributions of the phosphate and sugar moieties can disguise the adenine signals at such positions. The bands at 1045 cm^−1^ at point 3 and 1039 cm^−1^ at position 4 are assigned to the N–sugar stretching and C–O sugar stretching vibrations and the bands at 1068 cm^−1^ at position 3 and 1072 cm^−1^ at position 4 are assigned to the N–sugar stretching and PO_2_^−^ stretching vibrations. This strongly indicates an interaction of the backbone of the DNA strands with the silver tip and agrees well with the previously mentioned formation of loops along the strand.

A Raman band around 725 cm^−1^ is usually present in spectra of purine derivates (except guanine), and this is attributed to the pyrimidine ring breathing mode. Pure adenine shows this band at 723 cm^−1^ and poly(dA) at 727 cm^−1^. In the TERS spectra the previously mentioned binding of one or more nitrogen atoms to silver atoms of the tip, and/or conformational changes, cause a shift to 733 cm^−1^.

**Figure 3:**
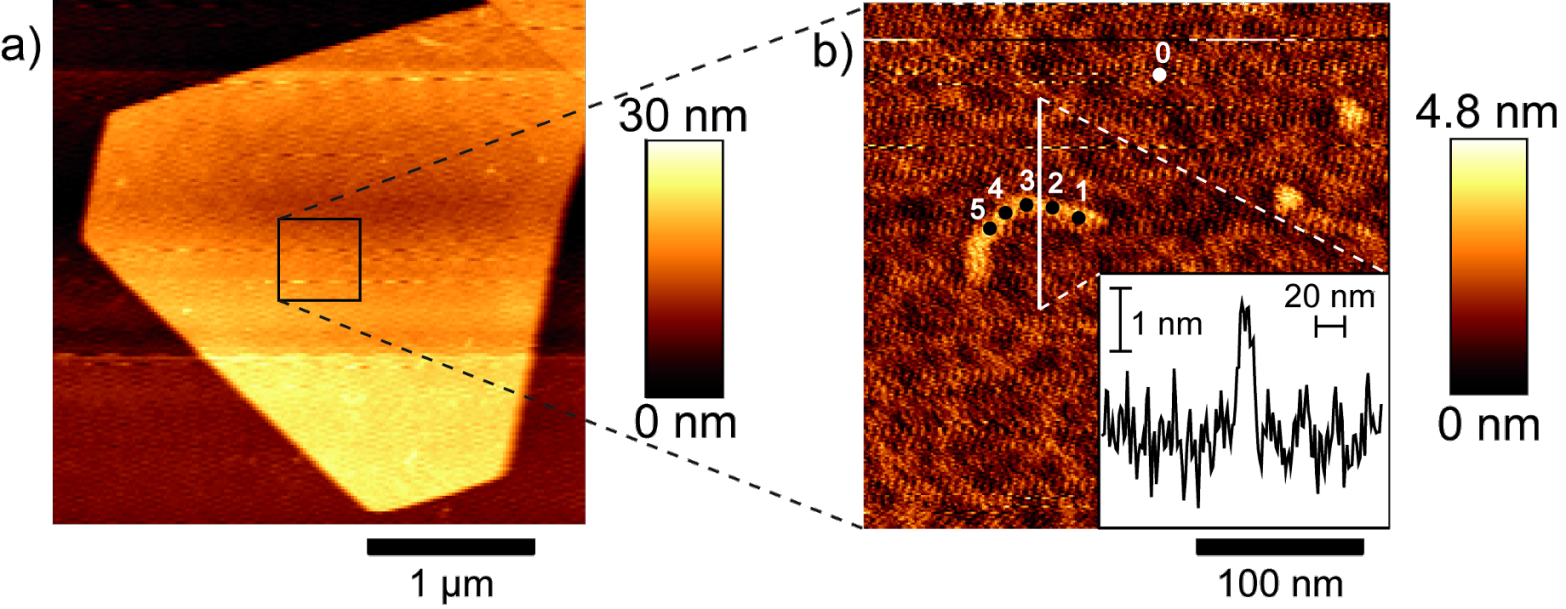
a) AFM topography image of a gold nanoplate with immobilized polyuracil strands, b) single strand of uracil homopolymer in the magnified area indicated in a). Inset in b) is the cross section corresponding to the line indicated in b).

In order to test the feasibility of different sample supports and to further check the reproducibility of the setup, TERS experiments on uracil homopolymer strands on transparent ultraflat gold substrates were performed. Figure 3 shows the AFM topography of a uracil strand immobilized on a typical gold nanoplate substrate. The magnification of the indicated area, in particular the height profile, indicates a single strand of polyuracil.

**Figure 4:**
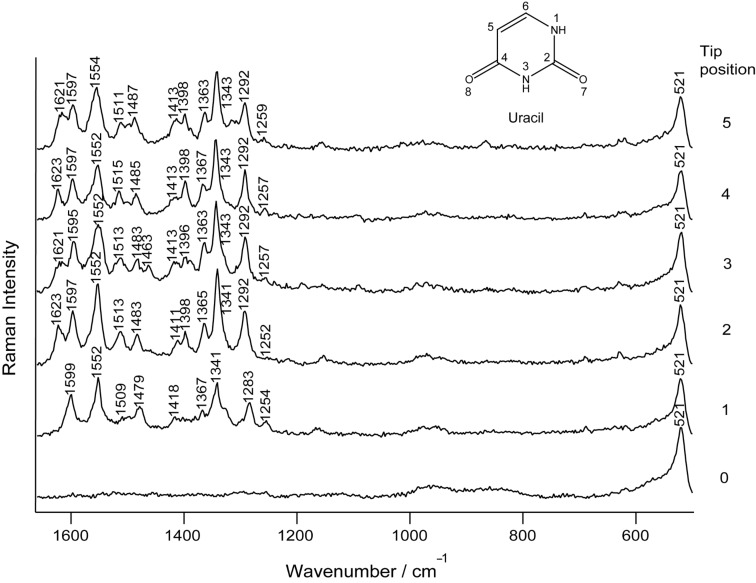
TERS spectra of the uracil homopolymer, measured at the positions indicated in Figure 3b.

TERS spectra were obtained on five different positions on the strand and are shown in Figure 4. In addition a reference spectrum was recorded on pure gold to exclude spurious results due to tip contamination.

The spectra look remarkably similar and the main spectral features of uracil can be assigned. Minor variations in band intensity ratios and positions can be attributed to the previously discussed effects that appear when measuring with such a high lateral resolution [30]. Surprisingly, the usually intense ring breathing mode of uracil at 800 cm^−1^ is not visible in the spectra. As those vibrations that involve polarizability changes parallel to the TERS tip axis are expected to be more enhanced than perpendicular vibrations, the lack of the ring breathing mode may be due to a flat orientation of the ring on the gold surface. Similar effects were found to influence the ring breathing mode of aromatic amino acids [31]. Apart from this difference the spectral features can be attributed solely to uracil and no signals of the sugar and phosphate backbone were found. This suggests a strong immobilization of the homopolymer on the gold surface through the phosphate backbone. This finding, however, somewhat contradicts the idea of a flat orientation of the ring with respect to the gold substrate, and no explanation can be given at the moment.

An assignment of the measured spectra is provided in Table 2.

**Table 2:** Band assignment of the TERS spectra of the uracil homopolymer (cm^−1^).^a^

1	2	3	4	5	Assignment

521	521	521	521	521	silicon (AFM tip)
1254	1252	1257	1257	1259	str N_3_C_4_, bend N_1_H, C_5_H, C_6_H [32]
1283	1292	1292	1292	1292	str N_3_C_4_ (–C_4_C_5_–C_6_N_1_), bend N_1_H, C_5_H, C_6_H [33]
1341	1341	1343	1343	1343	bend N_3_H, C_5_H, C_6_H, str C_2_N_3_ [33–34]
1367	1365	1363	1367	1363	str N_1_C_2_–C_2_N_3_ + C_4_C_5_ (–C_2_O_7_), bend C_5_H [32]
	1398	1396	1398	1398	bend N_1_H, C_5_H, C_6_H [32]/str (NC–CN + CC–CN) [35]
1418	1411	1413	1413	1413	bend N_3_H, N_1_H, str N_1_C_2_, N_3_C_4_ [34]
		1463			bend C_6_H, N_1_H, N_2_H, C_5_H, str C_4_C_5_ [34]
1479	1483	1483	1485	1487	str C_6_N_1_–C_4_C_5_–C_2_O_7_, bend N_1_H, C_5_H, C_6_H [32]
1509	1513	1513	1515	1511	bend N_1_H, str C_6_N_1_ [34]/def (NH) + in-plane ring str [35]
1552	1552	1552	1552	1554	str C_4_O–C_5_C_6_–C_2_O, bend N_1_H, C_6_H [33]
1599	1597	1595	1597	1597	str C_4_O_8_, bend N_1_H, C_6_H [32]
	1623	1621	1623	1621	str C_2_O_7_ + C_5_C_6_, bend N_1_H [32]

^a^Abbreviations: str, stretching; bend, bending; def, deformation.

**Figure 5:**
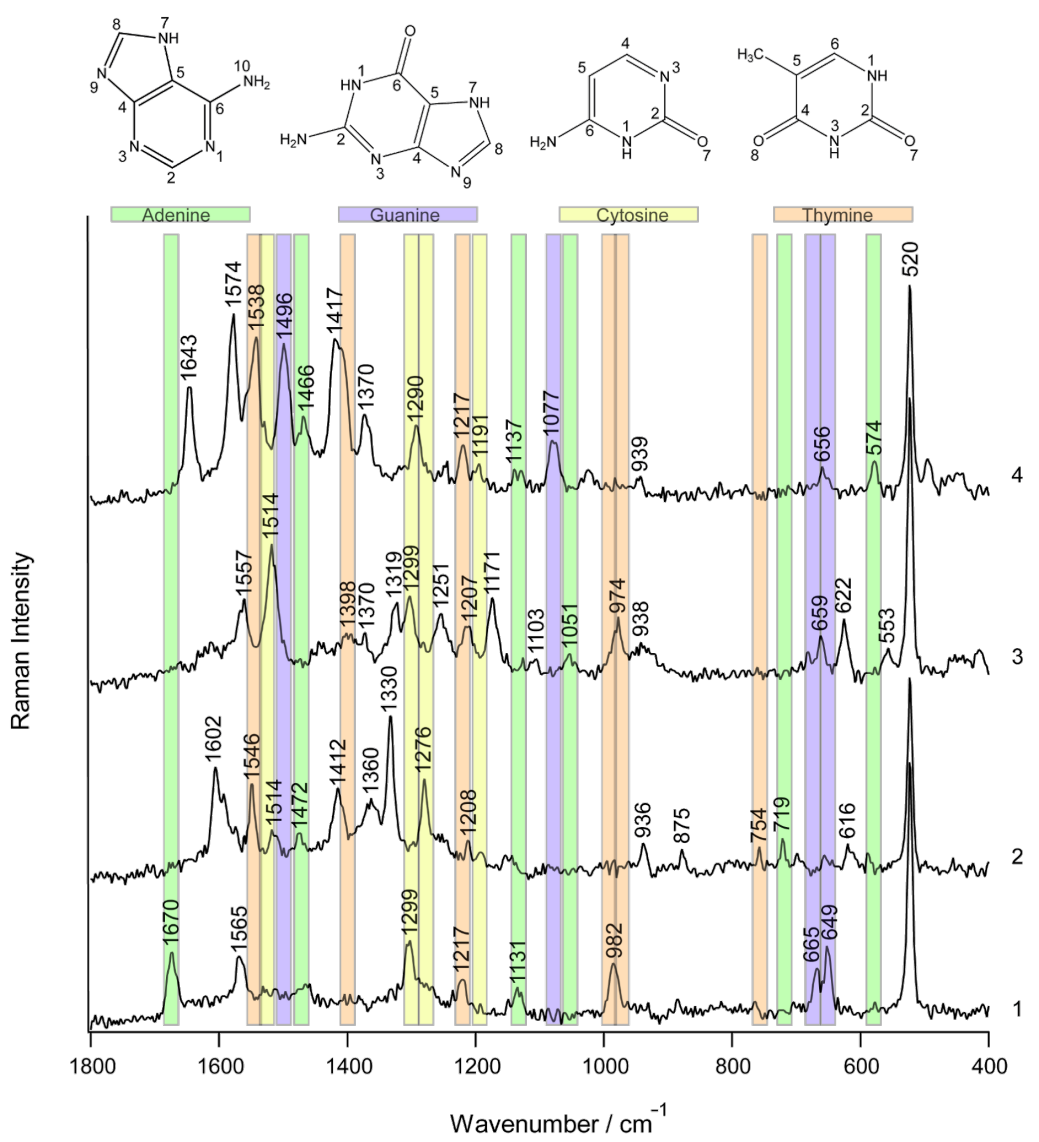
TERS spectra measured at four positions on single stranded calf thymus DNA. Unambiguously assignable bands are highlighted.

In a further step we address the issue of the different Raman cross sections of the distinct bases. Figure 5 shows four representative TERS spectra, out of a series of 26, that were measured on single stranded calf thymus DNA immobilized nonspecifically on a mica substrate. Spectral features of adenine, cytosine, guanine and thymine can be seen in the spectra. A tentative assignment of these four data sets is given in Table 3. As expected, when compared to the previously shown data on homogeneous strands, the spectra show a striking difference when measured at distinct tip positions, a fact that can be related to specific nucleobase patterns underneath the tip.

**Table 3:** Band assignment of the TERS spectra of the calf thymus DNA (cm^−1^).^a^

1	2	3	4	Assignment

520	520	520	520	silicon (AFM tip)
		553		C/T (ring def) [35]
			574	A (C_2_–H, N_9_–H wag) [35]
	616	622		A/T (ring def) [35]
649		659	656	G (ring breathing) [15,24]
665				G (ring breathing (Im)) [27]
	719			A (ring breathing) [15,35–36]
	754			T (ring breathing) [35,37]
	875			deoxyribose ring [38]
	936	938	939	A/C/G (NH_2_ rk ) [27,35]/deoxyribose [39]
		974		T (CC str, CO str, ribose) [40]
982				T (out-of-plane NH_2_ wagging) [35]
		1051		A (N–sugar str) [27]
			1077	G [27]
		1103		PO_2_ sym str
1131			1137	A (C_8_–N_9_ str, N_9_–H, C_8_–H def) [35]
		1171		A/G (C_5_–C_6_ str) [27]
			1191	C [15]
	1208	1207		T (ring str) [15,27,40]
1217			1217	T (in-plane ring-CH_3_ str) [35]
		1251		A [35,37]/C [37]/G (C_8_–N_9_ str) [27]/T (ring str) [35]
	1276			C (C–NH_2_ str + ring str) [35,39]
1299		1299	1290	C (C_2_–N_3_ str) [24,27]
		1319		A/G (C–N str (Im)) [27]
	1330			A/G (ring mode) [38]
	1360	1370	1370	A/C/G/T (C–N str (Py)) [27]
		1398		T (NH def/CH_3_ def) [27,35]
	1412		1417	C (C_4_–C_5_ str) [27]/A [35]/T [35]
	1472		1466	A (C=N str (Py)) [27]
			1496	G (C=N str (Im)) [27]
	1514	1514		C (NH_2_ def) [27]
	1546		1538	T (in-plane ring str) [35]
1565		1557	1574	A/C/G/T (ring str (Py)) [27]
	1602			A/C/G (NH_2_ def) [27,37]
			1643	C/G/T (C=O str, C=C str) [27,40]
1670				A (NH_2_ sci) [27]

^a^Abbreviations: Py, pyrimidine; Im, imidazole; str, stretching; def, deformation; rk, rocking; sci, scissoring.

Among the 26 collected spectra, five show nucleobase ring breathing modes and no contributions from phosphate or ribose, indicating an immobilization of the DNA strand on the mica substrate through the phosphate backbone. The adenine ring breathing mode is shifted to lower wavenumbers due to the deformation of the molecule by the silver tip, supposedly because the silver atoms pushed against the N_3_ nitrogen during the measurement [16]. The guanine ring breathing mode is down shifted as well, and thus it is likely that the same effects are responsible for this observation. Seven spectra showed spectral contributions from the phosphate and sugar, and no ring breathing modes were detected. Consequently in these cases the nucleobases are preferentially orientated perpendicular to the TERS tip axis, i.e., flat on the substrate surface, and only the DNA backbone interacts with the silver tip. Finally some TERS spectra show bands that can be attributed to ring breathing modes, to the ribose or to the phosphate backbone, as for example spectrum 3, either pointing towards a nonspecific binding of the DNA strands or to a coiled structure.

Assigning the bands of the TERS spectra is challenging regarding the different proposed band assignments found in the literature. An example is the band at 1251 cm^−1^ in spectrum 3. It can be attributed to the (C_8_–H, N_9_–H) deformation, the (N_7_–C_8_) stretching of adenine [35], a ring mode of adenine or cytosine [37], the (C_8_–N_9_) stretching of guanine [27] or the in-plane ring stretching of thymine [35].

Nevertheless, despite these difficulties and the previously mentioned intrinsic properties of the TERS setup, most bands can be unambiguously assigned to a specific base and thus can serve as marker bands. Others, such as the band at around 938 cm^−1^ in spectra 2, 3 and 4, cannot be related to a distinct base, but instead can be assigned to the NH_2_ rocking mode of adenine, cytosine and guanine and, hence, exclude thymine. The band at around 650 cm^−1^ can be assigned to the guanine ring breathing mode. It usually is more intense, but it is also polarization and orientation dependent. Green et al. reported the detection of two Raman bands at 650 cm^−1^ and 688 cm^−1^, both associated with the guanine ring breathing mode [24]. As the normal Raman band of free guanine is found at 680 cm^−1^ and the SERS band at 650 cm^−1^, different adsorption configurations can be assumed, while the appearance of both bands in one spectrum of DNA can be explained by a steric influence of the backbone on the adsorption behavior of the strands. In our case the two bands never appeared in one spectrum and only once was the guanine band at 686 cm^−1^ detected.

Adenine exhibits a higher Raman scattering cross section than guanine. The band at 1319 cm^−1^ in spectrum 3 and the band at 1330 cm^−1^ in spectrum 2 can be attributed to adenine and guanine, respectively. To support this assignment further information from the other spectra must also be considered. Spectrum 3 shows the guanine ring breathing mode; consequently the band at 1319 cm^−1^ can be assigned to guanine. In spectrum 2 the adenine ring breathing mode appears (719 cm^−1^), supporting the assignment of the band at 1330 cm^−1^ to adenine.

The band at 754 cm^−1^ in spectrum 2 refers to the ring breathing mode of thymine [35,37], but this mode also exhibits a band at around 800 cm^−1^ [15,18,35]. In our case only one of these bands was detected in the respective TERS spectra. This can probably be explained by orientational effects, similarly to the case of adenine.

The previously mentioned different Raman scattering cross sections of the nucleobases are reflected in the TERS spectra to a certain extent, as there are more spectral contributions from adenine, guanine and cytosine and relatively little from thymine. However, evidence for all four nucleobases was found, and consequently a sequence-specific detection is only restricted by the lateral resolution and ultimately by the signal-to-noise ratio of the spectra recorded at subsequent positions.

## Conclusion

Our results present several important steps towards a direct and label-free sequencing of RNA/DNA strands. First of all a reproducible immobilization of DNA and RNA strands on different substrates could be achieved. The successful TERS measurement on a uracil homopolymer immobilized on a gold nanoplate substrate is of particular interest regarding the additional field enhancement and field confinement attributed to a metal substrate. The TERS spectra collected on the DNA and RNA homopolymers show a high reproducibility of the spectral features of the respective base and clearly demonstrate the effects related to the tip location. A very important finding was that spectral contributions from all four nucleobases can be detected and distinguished on a genuine strand. In previous experiments it was shown that the limits of detection of TERS already reach single base sensitivity [19]. In combination with the results presented here, this means that when the TERS probe is laterally shifted over the DNA strand in intervals of less than one base-to-base distance the subsequent spectra provide the sequence information.

## Experimental

### Sample preparation

A single stranded DNA homopolymer of adenine (Sigma-Aldrich Chemie GmbH, Germany), a single stranded RNA homopolymer of uracil (Sigma-Aldrich) and single stranded calf thymus DNA (Sigma-Aldrich) were used in these experiments without further purification. Any further chemicals used for buffer solutions and colloid synthesis were purchased either at Sigma-Aldrich or VWR international. The adenine homopolymer and the calf thymus DNA were dissolved in a buffer solution consisting of HEPES (20 mM, 4-(2-hydroxyethyl)-1-piperazineethanesulfonic acid) and magnesium chloride (20 mM, MgCl_2_) to immobilize the strands through the negatively charged phosphate backbone by means of divalent cations (Mg^2+^) on mica (BAL-TEC) [19]. The concentration was 10–100 ng/µL. 1 µL of the respective solution was dropped on a freshly cleaved mica sheet and incubated under an argon atmosphere. Residues from the buffer were removed prior to the TERS measurements by rinsing with double distilled water and subsequent drying under argon [41].

The uracil homopolymer was dissolved in water and immobilized through nonspecific adsorption through the pyrimidine ring on gold nanoplates. The synthesis is described in detail in [22]. For the immobilization procedure the gold nanoplates were centrifuged onto a clean cover slide. 1 µL of the polyuracil solution was dropped on this substrate and incubated under an argon atmosphere. Excess RNA strands were removed with double distilled water and subsequent drying under argon.

### Instrumentation

The TERS setup used for the experiments on the adenine homopolymer and the calf thymus DNA has been described in detail elsewhere [15,26,42]. For all TERS spectra of the adenine homopolymer the laser intensity on the sample was set to 500 µW at 530.9 nm and the acquisition time was 5 s. For all TERS measurements on the calf thymus DNA the laser intensity at the sample was set to 1 mW at 568.2 nm and the acquisition time was 10 s.

The TERS setup employed for the measurement on the uracil homopolymer consists of an adapted Raman spectrometer (Acton Advanced SP2750 A, S&I GmbH, Germany) in combination with an AFM (NanoWizard II, JPK Instruments AG, Germany). The laser intensity on the sample was set to 860 µW at 532 nm and the acquisition time was 5 s.

Regarding both instruments, the time lag between two spectra is <1 second, which corresponds to the tip moving to the next position and the storage of the acquired data.

## References

[1] Maxam A M, Gilbert W (1977). Proc Natl Acad Sci U S A.

[2] Sanger F, Nicklen S, Coulson A R (1977). Proc Natl Acad Sci U S A.

[3] Treffer R, Deckert V (2010). Curr Opin Biotechnol.

[4] Stöckle R M, Suh Y D, Deckert V, Zenobi R (2000). Chem Phys Lett.

[5] Hayazawa N, Inouye Y, Sekkat Z, Kawata S (2000). Opt Commun.

[6] Anderson M S (2000). Appl Phys Lett.

[7] Hartschuh A, Sánchez E J, Xie X S, Novotny L (2003). Phys Rev Lett.

[8] Pettinger B, Picardi G, Schuster R, Ertl G (2002). Single Molecules.

[9] Schmid T, Messmer A, Yeo B-S, Zhang W, Zenobi R (2008). Anal Bioanal Chem.

[10] Cialla D, Deckert-Gaudig T, Budich C, Laue M, Möller R, Naumann D, Deckert V, Popp J (2009). J Raman Spectrosc.

[11] Neugebauer U, Rösch P, Schmitt M, Popp J, Julien C, Rasmussen A, Budich C, Deckert V (2006). ChemPhysChem.

[12] Böhme R, Richter M, Cialla D, Rösch P, Deckert V, Popp J (2009). J Raman Spectrosc.

[13] Böhme R, Cialla D, Richter M, Rösch P, Popp J, Deckert V (2010). J Biophotonics.

[14] Yeo B-S, Stadler J, Schmid T, Zenobi R, Zhang W (2009). Chem Phys Lett.

[15] Rasmussen A, Deckert V (2006). J Raman Spectrosc.

[16] Watanabe H, Ishida Y, Hayazawa N, Inouye Y, Kawata S (2004). Phys Rev B.

[17] Domke K F, Zhang D, Pettinger B (2007). J Am Chem Soc.

[18] Zhang D, Domke K F, Pettinger B (2010). ChemPhysChem.

[19] Bailo E, Deckert V (2008). Angew Chem, Int Ed.

[20] Yang Z, Aizpurua J, Xu H (2009). J Raman Spectrosc.

[21] Futamata M, Maruyama Y, Ishikawa M (2003). J Phys Chem B.

[22] Deckert-Gaudig T, Deckert V (2009). Small.

[23] Deckert-Gaudig T, Erver F, Deckert V (2009). Langmuir.

[24] Green M, Liu F-M, Cohen L, Köllensperger P, Cass T (2006). Faraday Discuss.

[25] Barhoumi A, Zhang D, Tam F, Halas N J (2008). J Am Chem Soc.

[26] Budich C, Neugebauer U, Popp J, Deckert V J (2008). Microsc.

[27] Jang N-H (2002). Bull Korean Chem Soc.

[28] Otto C, De Mul F F M, Huizinga A, Greve J (1988). J Phys Chem.

[29] Movileanu L, Benevides J M, Thomas G J (1999). J Raman Spectrosc.

[30] Deckert-Gaudig T, Bailo E, Deckert V (2008). J Biophotonics.

[31] Deckert-Gaudig T, Rauls E, Deckert V (2010). J Phys Chem C.

[32] Cho K-H, Choo J, Joo S-W (2005). Spectrochim Acta, Part A: Mol Biomol Spectrosc.

[33] Lin Z-B, Xie B-G, Tian J-H, Tang Y-A, Sun J-J, Chen G-N, Ren B, Mao B-W, Tian Z-Q (2009). J Electroanal Chem.

[34] Giese B, McNaughton D (2002). J Phys Chem B.

[35] Badr Y, Mahmoud M A (2006). Spectrochim Acta, Part A: Mol Biomol Spectrosc.

[36] Giese B, McNaughton D (2002). J Phys Chem B.

[37] Ke W, Zhou D, Wu J, Ji K (2005). Appl Spectrosc.

[38] Thomas G J, Benevides J M, Overman S A, Ueda T, Ushizawa K, Saitoh M, Tsuboi M (1995). Biophys J.

[39] Hennemann L E, Meixner A J, Zhang D (2010). Spectroscopy.

[40] Escobar R, Carmona P, Molina M (1996). Analyst.

[41] Hansma H G, Revenko I, Kim K, Laney D E (1996). Nucleic Acids Res.

[42] Bailo E, Deckert V (2008). Chem Soc Rev.

